# Impact of body mass index on fracture severity, clinical, radiological and functional outcome in distal radius fractures: a retrospective observational study after surgical treatment

**DOI:** 10.1007/s00402-024-05391-6

**Published:** 2024-05-30

**Authors:** Anna Lena Kloberdanz, Jasmin Meyer, Kora Kammermeier, André Strahl, Carsten Schlickewei, Konrad Mader, Karl-Heinz Frosch, Sinef Yarar-Schlickewei

**Affiliations:** Department of Trauma and Orthopedic Surgery, University Medical Center Hamburg-Eppendorf, Martinistr. 52, 20246 Hamburg, Germany

**Keywords:** Distal radius fracture, Body-Mass-Index, DASH, PROM, Clinical outcome, Radiological outcome

## Abstract

**Introduction:**

Distal radius fracture (DRF) is one of the three most common fractures of the human body with increasing incidences in all groups of age. Known causes of increasing incidence, such as ageing of the population or increased obesity, have been described and discussed. So far, literature reports ambivalent effects of body mass index (BMI) on bone physiology. It is worthwhile to examine the influence of BMI on the outcome of fractures more detailed. This study aims to investigate the influence of an abnormal BMI on fracture severity and treatment, as well as clinical, radiological, and functional outcome to improve clinical decision making.

**Materials and methods:**

A retrospective observational study was conducted on data obtained from patients, who underwent open reduction and internal fixation (ORIF) of a DRF at a local Level 1 Trauma Center between May 2018 and October 2021. Follow-up examinations were performed approximately 1 year after surgical fracture treatment, during which various questionnaires and functional measurements (CMS, DASH, NRS, ROM) were applied. In addition, postoperative complications were recorded and radiological examinations of the affected hand were performed. After excluding incomplete data sets and applying set exclusion criteria, the complete data of 105 patients were analyzed.

**Results:**

74 patients were female and 31 male with significant difference in mean BMI [*p* = 0.002; female: 23.8 (SD ± 3.3), men: 26.2 (SD ± 3.9)]. Patients with higher BMI had significantly more severe fractures (*p* = 0.042). However, there was no significant difference in surgery time for fracture management. At follow-up, patients with lower BMI showed a smaller difference in hand strength between the fractured and the other hand (*p* = 0.017). The BMI had no significant effect on the clinical and radiological outcome.

**Conclusion:**

Despite the ambivalent effects of BMI on the skeletal system, our findings indicate that a higher BMI is associated with more severe DRF. Thereby BMI does not correlate with surgery time for fracture treatment. Furthermore, no evidence of an influence on the clinical and radiological outcome could be detected.

**Supplementary Information:**

The online version contains supplementary material available at 10.1007/s00402-024-05391-6.

## Introduction

Distal radius fracture (DRF) occurs in Germany with an incidence of 106 per 100,000 per year, making it one of the three most common fractures of the human musculoskeletal system. The treatment is an established part of everyday clinical practice in trauma surgery [1]. Two peaks in prevalence can be identified. One around the age of 10 and one at older ages around the age of 60 [2]. However, the incidence of DRF appears to be increasing in all groups of age. Different causes for this development are discussed [3]. It is known that environmental influences as adverse weather conditions and patient-related factors such as age, gender, and lifestyle have an impact on the incidence of DRF [4, 5]. Women are significantly more affected [1]. In this regard, lifestyle has a crucial influence on patients’ body weight. Both overweight and underweight are not only a risk factor for cardiometabolic diseases, but also significantly increase the risk for musculoskeletal diseases such as osteoporosis [6, 7].

The body mass index (BMI) has gained global acceptance as a valuable tool for evaluating and assessing underweight and overweight individuals. The classification of BMI for adults introduced by the World Health Organization (WHO) in 1995 is generally used in daily clinical practice. Simplified, the following categories are distinguished: BMI ≥ 30 kg/m^2^ obese, BMI > 25 kg/m^2^ overweight, 24.9–18.5 kg/m^2^ normal weight, < 18.5 kg/m^2^ underweight [8]. The influence of BMI on the risk of fracture is complex. It differs depending on the skeletal region and the associated bone mineral density. While an increased risk of hip and humerus fractures has been described for low BMI, a reduced risk has been described for distal forearm fractures, osteoporotic fractures and tibia and fibula fractures. An increased BMI was associated with an increased risk of humerus fractures and osteoporotic fractures [9]. With an increased BMI, in addition to putative benefits such as a protective soft tissue mantle or increased bone strength, greater mechanical stress on bone have been reported [10]. Underweight or lowered BMI is associated with soft tissue loss, muscle weakness (increased risk of falls), and often malnutrition [7]. As a result, altered bone structure and decreased bone mass or strength, which occurs in both overweight and underweight people, may lead to an increased risk of fracture [11].

Surgical treatment of DRF is an established procedure [12]. Several plate designs are available for surgical therapy. It is important to investigate whether BMI has an influence on the outcome or on the time of surgery. Recent studies have shown, that an elevated BMI leads to an elongated time of surgery. This has implications for cost and efficiency in the operating theatre [13]. In addition, longer operating times can increase morbidity and the risk of infection.

In summary, the purpose of this study is to investigate the influence of an abnormal BMI on fracture severity and surgery time in DRF using a retrospective cohort. All patients in this study received clinical and radiological re-examination after a 1-year follow-up and were rated using patient reported outcome measures (PROMs). The aim is to capture a comprehensive assessment of the impact of abnormal BMI on fracture entity, surgical challenges and healing process of a DRF.

## Materials and methods

Ethics committee (Ethikkommission der Hamburger Ärztekammer) approval was given for retrospective registration (Reference number: WF-114/20). We confirm that all methods were performed in accordance with the relevant guidelines and regulations.

### Study cohort

**Fig. 1:**
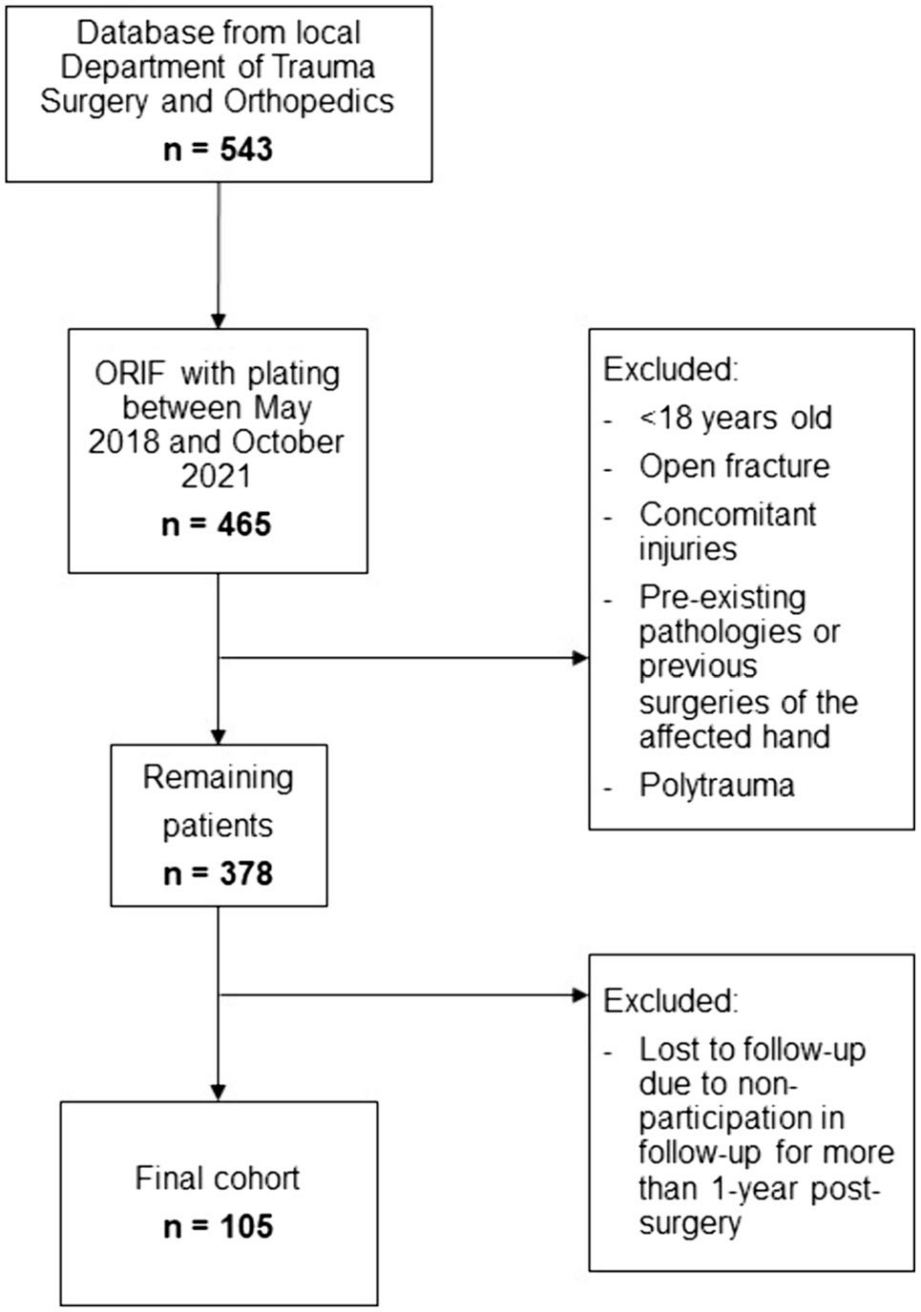
Flowchart. Retrospective selection of the study population, consisting of patients with distal radius fractures who received ORIF with locking plates in our department. The patients were enrolled in follow-up examinations approximately 1 year after

A retrospective observational study was conducted of all patient data from the local Level 1 Trauma Center who underwent ORIF of a DRF with locking plates between May 2018 and October 2021. Patients were excluded if they were under 18 years of age, had an open fracture, or previous surgery on the affected hand. Polytrauma patients were also excluded. All patients were examined one year after surgical treatment of the DRF. The distribution of dorsopalmar plate osteosynthesis were balanced in both groups and did not show a significant difference. All patients included in the study received the same standardized postoperative therapy regimen: wearing a self-removable wrist orthosis for 6 weeks after the operation, immediate start of physiotherapy—extension/flexion without strain and avoiding pronation/supination and weight-bearing activities. After 6 weeks, the wrist can be moved freely. Thus, our cohort consists of 105 patients with 108 DRFs who remained after application of the exclusion criteria and with complete adherence to the follow-up examinations (Fig. 1).

### Patient-specific data

At time of diagnosis, demographic data (age, sex, height, weight), as well as fracture-related data (handedness, fracture side, concomitant wrist injuries and preoperative imaging) were obtained for each patient. Furthermore, the AO classification, including all subtypes, was determined and documented by the treating specialist and corrected by a senior surgeon. The BMI was calculated from the patient’s height and body weight. For better comparability of the results, the cohort was divided into two subgroups: BMI < 25: “no overweight” and BMI ≥ 25: “overweight”.

### Clinical and patient-reported outcome measures

At 1-year follow-up, clinical outcome was evaluated after fracture healing was complete. For this purpose, a clinical examination with measurement of range of motion (ROM) was performed. In addition, strength was measured on both hands using a hydraulic hand dynamometer (kg, Sammons Preston, model: Jamar®). Patients were additionally asked to complete the Disabilities of the Arm, Shoulder and Hand (DASH) questionnaire. It is used to subjectively assess functional disability in daily life [14, 15]. Patients were also asked to complete the Numerical Rating Scale (NRS). It was used to assess pain at rest and on exertion. Complications and postoperative sequelae were recorded, as well as the duration of surgery (incision—suture time). Finally, radiographic examination of the affected hand was performed at the local radiology department.

### Radiologic assessment

Two-plane radiographs were obtained immediately after surgical fracture treatment and at 1-year follow-up. The following measurements were obtained under the supervision of an attending physician: Radial height (mm), radial tilt (°), palmar tilt (°), ulnar variance (mm).

The 1-year radiograph was also used to assess complete consolidation of the fracture. The palmar plate position was evaluated using the Soong classification (Soong 0: dorsal to the watershed, Soong 1: volar to the watershed but proximal to the margin, Soong 2: volar to the watershed and on or distal to the volar margin) [16]. Furthermore, acceptable osteosynthesis was defined as ≤ 10° dorsal inclination, ≥ 15° radial inclination, < 2 mm ulnar variance, < 2 mm joint incongruence [17].

### Statistical analysis

Continuous variables are expressed as mean ± standard deviation (SD), while categorical variables are expressed as number and percentage The Shapiro–Wilk test was performed on all continuous variables to determine whether they were normally distributed. Based on the findings, either a parametric or non-parametric test was applied. To compare patients with an BMI < 25 and ≥ 25 in relation to continuous variables, Student’s t-test for independent samples was used for normally distributed data. For non-normally distributed data, the Mann–Whitney U test and for categorical variables, the Chi^2^ tests was applied. In order to compare the three AO classification subgroups, the one-way ANOVA for independent samples was conducted. SPSS statistical program 29.0 (SPSS, Chicago, IL) was used for all statistical analyses. The significance level was set at 0.05 for all statistical analyses. Exact p-values are reported unless *p* < 0.001.

## Results

### Demographics

The mean age within the cohort was approximately 59 years (± 16.1 years), with 74 (70.5%) patients being female and 31 (29.5%) patients being male. The majority of patients were right-handed and 6.7% were left-handed. 58% of patients had a BMI of < 25 (“no overweight”) and 42% had a BMI of ≥ 25 (“overweight”) (*p* = 0.083). Females having a mean BMI of 23.8 (± 3.3) and males of 26.2 (± 3.9) (*p* = 0.002). In addition to preoperative radiography, 78.7% of patients underwent computed tomography (CT), 4.6% underwent magnetic resonance imaging (MRI), and 2.8% patients underwent both CT and MRI. A detailed summary of the demographic data can be found in Table 1. Concomitant injuries were identified in approximately 52% of patients (Supp. Tab. 1).

**Table 1:** Demographic data of the cohorte

Characteristics	Value
Sex, n (%)	
Female	74 (70.5)
Male	31 (29.5)
Total	105 (100)
Age (yr)	
Mean (± SD)	59.2 (± 16.1)
Handedness, n (%)	
Right	98 (93.3)
Left	7 (6.7)
Body-Mass-Index (BMI), n (%)	
< 25	63 (58)
≥ 25	45 (42)
w, mean (± SD)	23.8 (± 3.3)
m, mean (± SD)	26.2 (± 3.9)
Fractured hands, n (%)	
Right	52 (48.1)
Left	56 (51.9)
Total	108 (100)
Preoperative imaging, n (%)	
X-ray	108 (100)
CT	85 (78.7)
MRI	5 (4.6)
CT and MRI	3 (2.8)
Concomitant injuries, n (%)	56 (51.9)

*yr.* years, *f* female, *m* male, *n (%)* results as absolute numbers and as percentage, *CT* computed tomography, *MRI* magnetic resonance imaging

*A detailed summary of each concomitant injury is provided in supplemental Table 1

### Fracture severity

**Fig. 2:**
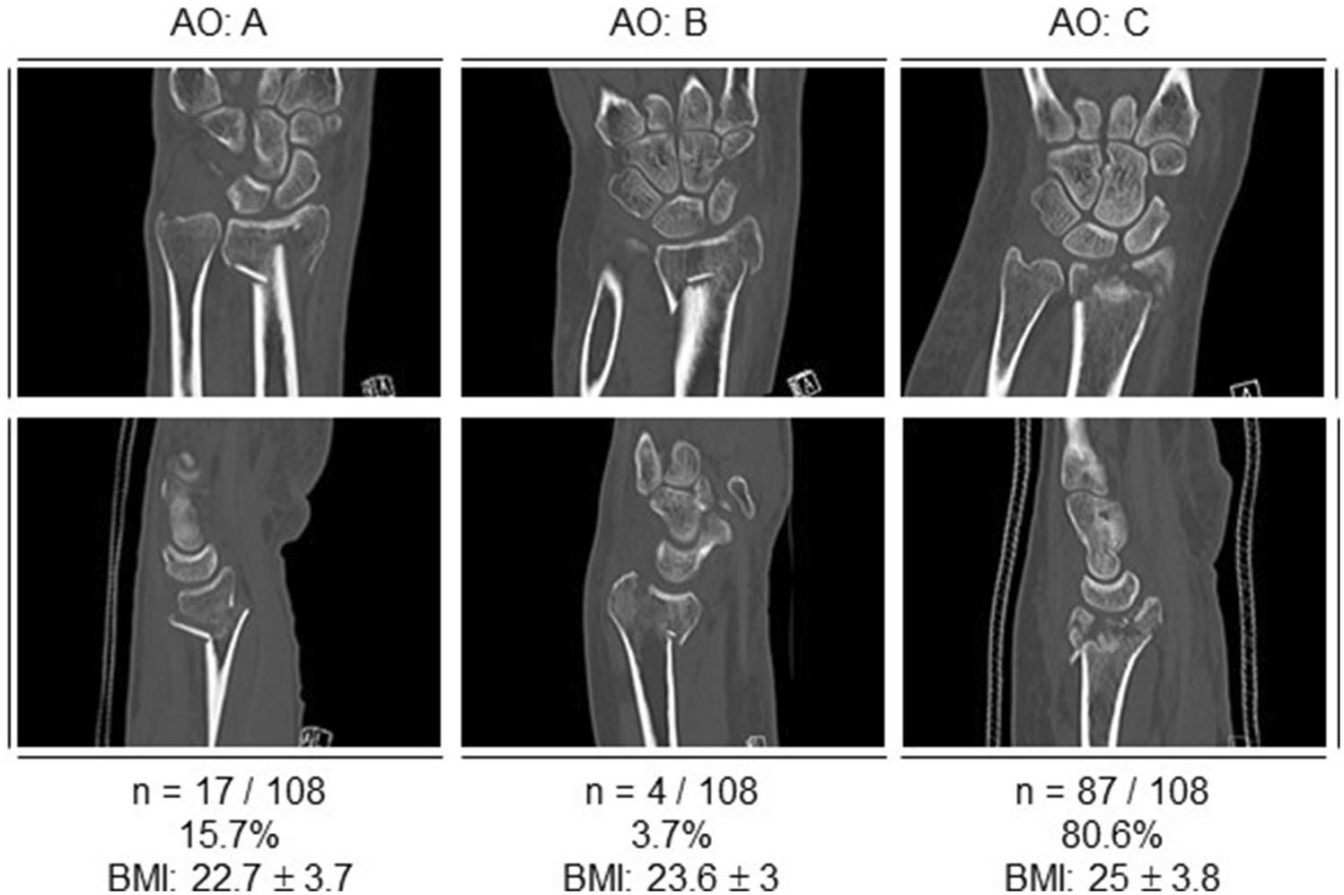
Summary of distal radius fractures according to the AO classification. Example CTScans for DRF with AO fracture type A, type B, and type C. Results are presented in absolute numbers and as a percentage (%) of the total cohort. BMI points per fracture type are expressed as mean ± standard deviation

According to the international AO classification, 15.7% of our cohort had a type A fracture, 3.7% had a type B fracture, and 80.6% had a type C fracture. The type A fracture group showed a mean BMI of 22.7 (± 2.1), type B fractures of 23.6 (± 3), and type C fractures of 25 (± 3.8) (Fig. 2). There was a significant difference in BMI between these three AO subgroups (*p* = 0.042). Accordingly, fracture severity increases with higher BMI. However, patients with a higher BMI did not show significantly more concomitant bony injuries than patients with a low BMI (*p* = 0.714).

### Surgery time

**Fig. 3:**
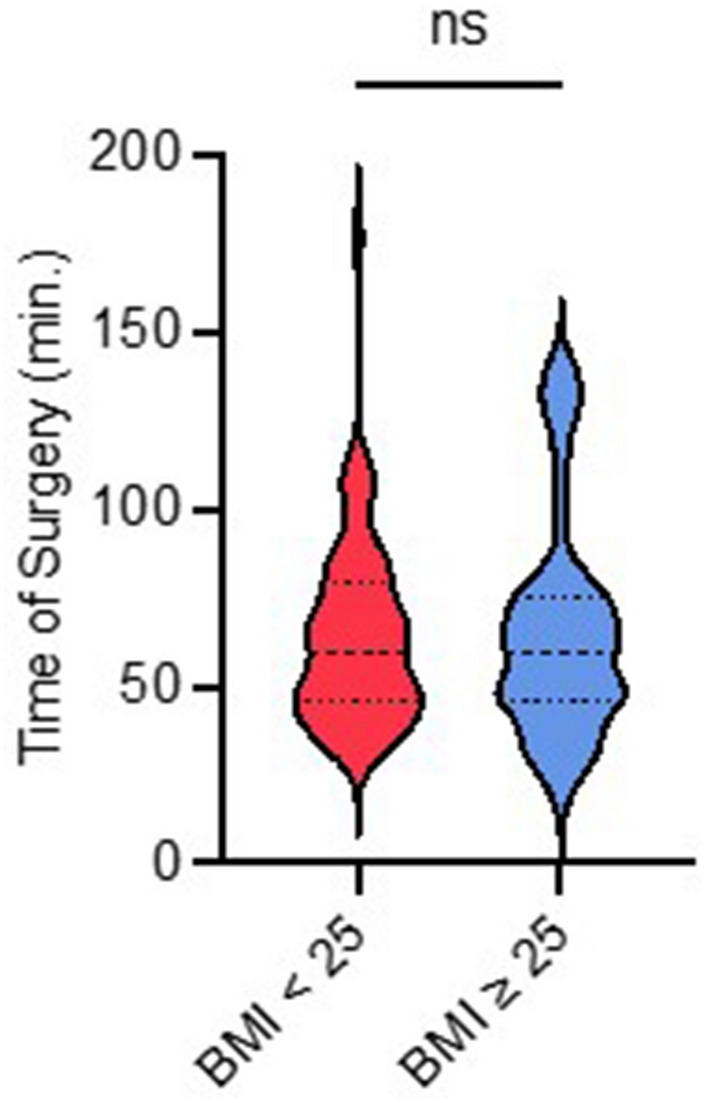
Association between duration of surgery and BMI. Analysis between the BMI subgroups (BMI < 25 “no overweight” and BMI ≥ 25 “overweight”) regarding the time of surgery, measured in minutes. ns: not significant (*p* = 0.497)

The average incision—suture time was 62.7 min (± 26.4 min; minimum: 20 min, maximum: 176 min). No significant difference regarding the duration of the operation was found between the two BMI subgroups (Fig. 3). Furthermore, there was no significant difference (*p* = 0.497) in incision—suture time for the treatment of a type A (55.6 ± 19.7 min), type B (45.8 ± 12.3 min), or type C fracture (64.8 ± 27.6 min).

### Clinical and patient-reported outcome measures

The follow-up examinations of all 105 patients took place after a mean of 18.2 months and a standard deviation of 7 months. No significant differences were found between the two BMI subgroups with respect to CMS testing, DASH score, NR scale pain, or foreign body sensation (Tab. 2). However, patients with a higher DASH score reported significantly more severe pain on the NRS (r = 0.279, *p* = 0.004).

**Table 2:** Comparison of scores on the various questionnaires and physical examinations of both hands at follow-up between the two BMI subgroups

Data collected at follow-up	BMI < 25	BMI ≥ 25	p-Value
CSM disorders, n (%)	13 (12)	14 (13)	0.215
DASH-score			
Mean (± SD)	9.9 (± 11.2)	11.8 (± 15.3)	0.475
Pain on Numeric Rating Scale (NRS)			
Rest, mean (± SD)	0 (± 1)	0 (± 1)	0.499
Exertion, mean (± SD)	2 (± 3)	2 (± 2)	0.129
Difference in grip strength between both hands (kg)			
Mean (± SD)	2.5 (± 4.8)	5.6 (± 7.0)	**0.017**
Foreign body sensation, n (%)	3 (6.7)	9 (14.3)	0.157
Peri-/Postoperative complications, n (%)	17 (15.7)	9 (8.3)	0.403
Difference in motion between both hands (°)			
Extension, mean (± SD)			
Active	7 (± 10)	8 (± 13)	0.546
Passive	8 (± 10)	10 (± 14)	0.390
Flexion, mean (± SD)			
Active	10 (± 12)	13 (± 12)	0.261
Passive	12 (± 14)	18 (± 14)	0.058
Ulnar deviation, mean (± SD)			
Active	2 (± 8)	4 (± 11)	0.313
Passive	2 (± 9)	4 (± 12)	0.356
Radial deviation, mean (± SD)			
Active	2 (± 8)	1 (± 11)	0.876
Passive	3 (± 9)	0.1 (± 12)	0.198
Supination, mean (± SD)			
Active	2 (± 7)	5 (± 11)	0.172
Passive	2 (± 7)	4 ± 14	0.371
Pronation, mean (± SD)			
Active	1 (± 4)	1 (± 4)	0.964
Passive	2 (± 6)	0.3 (± 5)	0.153

Hand strength showed a significant difference with a mean of 21.5 kg (± 9.97 kg) for the surgically treated hands and 25.4 kg (± 9.5 kg) for the unaffected hands, respectively (*p* < 0.001). Patients with higher DASH scores also had significantly lower hand strength in the operated hand (*p* < 0.001) compared to the unaffected hand. When comparing the difference in hand strength between the operated and unaffected hand in the BMI subgroups, the difference in hand strength was significantly less in the "no overweight" BMI subgroup (2.5 kg ± 4.8 kg) compared to the "overweight" subgroup (5.6 kg ± 7.0 kg) (*p* = 0.017). 24.1% (n = 26/108) of all operated hands showed peri- or postoperative complications. They manifested as tendon or nerve injury, revision surgery due to inadequate reduction or implant malpositioning, complex regional pain syndrome (CRPS), carpal tunnel syndrome, digitus saltans, and vascular injury. There was no significant difference in the complication rate between the BMI groups (“no overweight”: 15.7% and “overweight”: 8.3%). We found no significant difference in the ROM of the operated and non-operated hand of the BMI subgroups. The differences in each parameter between the operated and unoperated wrist in active and passive joint positions are shown in Table 2.

### Radiographic measurements

The osteosynthesis location was palmar in 92 cases (86.8%), dorsal in 7 cases (6.6%), and dorsopalmar in 7 cases (6.6%). The locking plates used were 97.2% Medartis AG and 2.8% VariAx™. The plate position was classified as Soong 0 in 19.2% of patients, Soong 1 in 19.2% of patients and Soong 2 in 61.6% of patients. All fractures were considered to be fully consolidated. In 25 patients, there was no evidence that the osteosynthesis was acceptable in terms to the mentioned criteria. This was due to radial tilt of < 15° in 20 patients, dorsal tilt of > 10° in three patients. In one patient, there was both a pronounced ulnar variance and a pronounced radial tilt. In one patient there was a missed diagnosis of SL ligament dissociation (SLAC III, DISI). We could not demonstrate a significant correlation between radiographic measurements and BMI (radial height: *p* = 0.336; radial inclination: *p* = 0.155; volar tilt: *p* = 0.378; ulnar variance: *p* = 0.547). Also, the changes in radiologic measurements between preoperative imaging and follow-up examination showed no significant difference among the BMI subgroups (Table 3).

**Table 3:** Comparison of changes in radiographic measurements at follow-up between the two BMI subgroups

Changes in radiographic measurements	BMI < 25	BMI ≥ 25	p-Value
Radial height (mm)			
Mean (± SD)	0.15 (± 1.2)	0.42 (± 1.6)	0.336
Radial inclination (°)			
Mean (± SD)	0.26 (± 2.5)	1.2 (± 3.4)	0.155
Volar tilt (°)			
Mean (± SD)	0.83 (± 4.7)	1.8 (± 6.5)	0.378
Ulnar variance (mm)			
Mean (± SD)	− 0.47 (± 1.7)	− 0.65 (± 1.4)	0.547

*mm* millimeter, (°) degree, *SD* standard deviation

## Discussion

This study analyzed clinical and radiological data from 105 patients with DRF to determine if there was an association between BMI and fracture severity, operative time, and clinical and radiological outcome. A significant relationship was found between BMI and fracture severity based on the AO classification. There seemed to be no effect of BMI on incision-suture time, clinical and radiological outcome. Functional outcome did not vary, except for a significantly smaller difference in hand strength in patients with lower BMI (“no overweight”).

For some other fractures than DRF, there is a consensus in literature postulating an increased BMI is associated with a significantly higher risk of a more severe fracture. For example, there is general agreement on the relationship between BMI and fracture severity when looking more closely at ankle and humerus fractures [9, 18].

For DRF, both Goodloe et al. and Montague et al. found a significant correlation between BMI and fracture severity. Both studies classified fracture severity according to the AO classification, as in this study. Goodloe et al. also reported an increased risk of intra-articular split fractures and intra-articular fragmentation [13]. Montague et al. demonstrated that the likelihood of a more complex DRF increased with each point increase in BMI [19]. In contrast, the study by Acosta-Olivo et al. did not show a significant increase in fracture severity, but did show a greater susceptibility to DRF in patients with higher BMI [20].

Some authors postulate that both a high BMI [21] and a low BMI protects against DRF. In their meta-analysis, Johansson et al. demonstrated a lower fracture rate in patients with a low BMI [9].

Similar to overweight, underweight is discussed in some studies as a risk factor for more severe forms of fracture. This is justified by cellular processes in bone metabolism that are affected by malnutrition and hormonal changes, and lean body mass [11]. Low BMI should therefore be discussed rather as a risk factor for more severe fractures [22–24].

A high BMI also influences the complex relationship between bone strain and bone strength. On the one hand, there are higher forces acting on the bone due to weight, which may explain the increase in fracture severity [25, 26]. On the other hand, higher body weight increases the mechanical stimuli on the bone, which enhances new bone growth and allows the bone to adapt to the increased body mass at the cellular level [27, 28]. However, this mechanism cannot be applied to the entire skeletal system.

The distal forearm, or the upper extremities in general, are not exposed to comparable mechanical stimuli as the lower extremities. For this reason, new bone formation on the distal forearm and the associated protective effect against fractures rather negligible.

Another protective mechanism mentioned in the literature is the soft tissue mantle formed by subcutaneous adipocytes, which may absorb impact forces and thus protect against fractures [29]. However, this cushioning does not provide uniform protection for all regions of the skeleton. In particular, at the forearm, the extent of the soft tissue mantle is very small and insufficient to reliably prevent the occurrence of fractures in general or to reduce the severity of fractures when a high force is applied [13, 29].

Thus, it can be concluded that increased body mass places greater stress on the bone, while protective mechanisms (bone strength and soft tissue cushioning) have little to no influence in DRF.

In addition to increased BMI, Ebinger et al. identified male sex and older age (over 50 years) as risk factors for complex fracture patterns [29]. The mean age of our cohort was 59 ± 16.1 years. It is possible that BMI and age may also have an additive effect on the likelihood of fracture severity.

Consistent with our findings, Zheng et al. describe optimal fracture prevention when both low and high BMI and early BMI loss can be avoided [30]. Our results underline that a BMI between 24.9–18.5 kg/m^2^, as recommended by the WHO, should be aimed for in order to avoid severe fractures [8].

An extension of surgery time of 0.38 min per BMI point has already been reported in the literature [13]. A similar correlation has been described in other surgical fields [31–34]. However, we were not able to demonstrate an increase in the duration of surgery due to a higher BMI. This may be due to the fact that there is less soft tissue surrounding the forearm. In addition, the treatment of an AO-A fracture took as long as the treatment of an AO-C fracture. Each operation could be performed with the same surgical efficiency regardless of BMI and fracture severity. In this way, cost inflation is controlled and staff capacity is conserved.

The complication rate of 24.1% of all patients that underwent surgery of distal radius fracture is comparable to other clinics. Other studies reported a postoperative complication rate of 39% after surgical treatment of distal radius fracture. The complications rate is increased due to a bias, as especially those patients presented to the follow-up who had complaints.

Common complications include tendinopathy, nerve injury, malposition, infection, healing in malposition, pseudarthrosis, chronic repetitive pain syndrome (CRPS) and compartment syndrome [35]. The complication rate had a high variability between different studies. McKay et al. found a complication rate varying from 6 to 80% [36]. Other studies have shown that obesity is associated with a higher risk for complications or revision surgery. In our study we were not able to show a significant association between the BMI and complication rate. The increased complication rate and rate for revision surgery in the study conducted by DeGeorge et al. was presented for patients with an BMI over 35 [37]. In our study we only analyzed the complication rate for patients with a BMI of over 25. The lower cut-off value of BMI in our study could explain the difference in both studies.

It is likely that increasing fracture severity with higher BMI also worsens functional outcome. However, in our cohort, all patients achieved good functional outcomes regardless of BMI. This fact has been confirmed in other studies [38, 39] and explains the high DASH score achieved by the patients in this study.

There was also no correlation between the radiological results and BMI. All fractures in our cohort are fully consolidated. We were not able to show that BMI has a negative effect on wound and bone healing processes in DRF or leads to a delay in these processes, as previously described for other fractures [40, 41]. This could be explained by the fact that we didn’t have an interim examination in which this was investigated.

This study is limited by the inaccurate estimation of the ratio of muscle to fat mass by BMI. More accurate results should be obtained in the future by detailed body fat measurement. This would also help to better classify the current health status of the patient. Another limitation is that especially those patients presented to the follow-up who had complaints. This could have led to a biased and increased complication rate. An additional limitation is the low number of patients included in the study, especially of those with an increased BMI.

Other parameters such as HBA1c should also be determined, as diabetes is also associated with impaired bone metabolism and a higher risk of osteoporosis. The risk of osteoporosis, and thus a correlation with BMI, could have been further supplemented with additional bone densitometry (DXA).

## Conclusion

This study shows that patients with a higher BMI have more severe distal radius fractures according to the AO classification. BMI does not correlate with surgery time for fracture management. Neither clinical nor radiological outcome is influenced by BMI.

### Supplementary Information

Below is the link to the electronic supplementary material.

Supplementary file1 (DOCX 12 KB)

## Data Availability

All relevant data is displayed in the figures/tables of this manuscript.
