# Supplementary material for: Impact of body mass index on fracture severity, clinical, radiological and functional outcome in distal radius fractures: a retrospective observational study after surgical treatment
Source: Arch Orthop Trauma Surg. 2024 May 30;144(6):2915–23. doi: 10.1007/s00402-024-05391-6 (PMC11211199; doi:10.1007/s00402-024-05391-6)
Supplement: Supplementary file 1 — Supplementary file1 (DOCX 12 KB) [file 402_2024_5391_MOESM1_ESM.docx]

**Supplemental Table 1**

| **Concomitant injuries (CI)** | **Value n (%)**  **(n = 56 patients)** |
| --- | --- |
| PSU avulsion | 50 (78.0) |
| Distal ulnar fracture | 7 (10.9) |
| SL ligament rupture | 2 (3.1) |
| Partial rupture FCR tendon | 1 (1.6) |
| N. medianus affection | 1 (1.6) |
| Avulsion of PCU | 1 (1.6) |
| Chip fracture Os triqu. | 1 (1.6) |
| Tear of middle finger capsular | 1 (1.6) |
| *Total CI* | *64 (100)* |
